# The Prognostic Impact of Unplanned Excisions in a Cohort of 728 Soft Tissue Sarcoma Patients: A Multicentre Study

**DOI:** 10.1245/s10434-017-5776-8

**Published:** 2017-01-20

**Authors:** Maria Anna Smolle, Per-Ulf Tunn, Elisabeth Goldenitsch, Florian Posch, Joanna Szkandera, Marko Bergovec, Bernadette Liegl-Atzwanger, Andreas Leithner

**Affiliations:** 1 Department of Orthopaedic Surgery, Medical University of Graz, Graz, Austria; 2 Sarcoma Centre HELIOS Klinikum Berlin-Buch, Berlin, Germany; 3 Orthopaedic Hospital Gersthof, Vienna, Austria; 4 Division of Clinical Oncology, Medical University of Graz, Graz, Austria; 5 Institute of Pathology, Medical University of Graz, Graz, Austria

## Abstract

**Background:**

Unplanned excisions (UE) of soft tissue sarcomas (STS) carry a high risk for local recurrence (LR) due to marginal/intralesional resections. However, there are reports about improved prognosis for UE patients who have re-resection compared with patients who undergo planned surgery. The present multicentre study was designed to define characteristics of UE patients and to investigate the impact of UE on subsequent therapy and patient outcomes.

**Methods:**

A total of 728 STS patients (376 males, 352 females; mean age: 58 years) who underwent definite surgery at one of three tumour centres were retrospectively included. Time-to-event analyses were calculated with log-rank and Gray’s tests, excluding patients with primary metastasis (*n* = 59). A propensity-score (PS) of being in the UE group was estimated, based on differences at baseline between the UE group and non-UE group. An inverse-probability-of-UE weight (IPUEW) was generated and time-to-event analyses calculated after IPUEW weighting.

**Results:**

Before referral, 38.6% of patients (*n* = 281) had undergone UE. Unplanned excision patients were younger (*p* = 0.036), rather male (*p* = 0.05), and had smaller (*p* < 0.005), superficially located tumours (*p* < 0.005). Plastic reconstructions (*p* < 0.005) and adjuvant radiotherapy (*p* = 0.041) more often were needed at re-resection. In univariable analysis, re-resected patients had improved overall survival (OS; *p* = 0.027) and lower risk of distant metastasis (DM; *p* = 0.002) than primarily resected patients, whereas risk of LR was similar (*p* = 0.359). After weighting for the IPUEW, however, differences in terms of OS (*p* = 0.459) and risk of DM (*p* = 0.405) disappeared.

**Conclusions:**

The present study does not support prior findings of improved outcome for UE patients. Unplanned excisions have a major impact on subsequent therapy, yet they do not seem to affect negatively the long-term oncology outcome.

Diagnosing soft tissue sarcomas among the great number of benign soft tissue lumps and bumps in routine practice is challenging. Soft tissue sarcomas are rare, accounting for only 0.7% of all malignancies estimated to be diagnosed in 2016 in the United States.^1^ Also, they often are asymptomatic or cause unspecific signs and symptoms. Therefore, STS patients are at risk of delayed or incorrect diagnosis.^2^ Because of these issues, unplanned excisions (UEs) are frequently performed. The proportion of patients referred following UE ranges between 18 and 53.3% of patients treated for STS at a sarcoma centre.^3^^–^ 5

Because UEs are usually the result of primary attempts to remove a lesion that is thought to be benign, they carry a high risk of residual tumour.^4^,^6^ Following UE, decision for or against re-resection, adjuvant radiotherapy (RTX), chemotherapy (CTX), or watchful waiting at the specialist centre is based on surgical and histological reports, resection margin status, and imaging taken prior to UE. Considering that marginal or even intralesional resection margins significantly increase the likelihood for local recurrence (LR), re-resections aiming at removal of residual tumour tissue often are justified.^6^

The combination of primarily inadequate resection, an unavoidable delay from UE to definite surgery, and the peril of residual tumour tissue all suggest that UE patients would do worse compared with initially adequately treated patients. Surprisingly, several study groups reported that UE patients have an identical or even better prognosis than patients who undergo planned surgery.^5^^–^ 7 It was concluded that patients with large and high-grade tumours at complicated anatomical sites are far more likely to be referred directly to a specialist centre, thus causing a selection bias. According to Lewis et al., however, even adjustment for these influential factors revealed a prognostic advantage for re-resected patients.^6^ The present multicentre study was designed to elucidate the baseline characteristics of UE cases, their impact on management at the specialist centre, and their potential influence on patient outcomes.

## Methods

### Patients

We included 728 patients undergoing surgery for STS between 1998 and 2015 at three sarcoma centres (Department of Orthopaedic Surgery, Medical University Graz; Orthopaedic Hospital Gersthof, Vienna; Sarcoma Centre, HELIOS-Klinikum Berlin-Buch). The mean patients’ age was 58 years (range 6–96 years; standard deviation (SD): ±16.9 years); 352 were female (48.4%) and 376 male (51.6%).

A standard template was used for data collection at each centre. UEs were defined as unintentionally performed resections of a subsequently histologically verified STS. Original tumour size was ascertained from preoperative MRI and/or medical records or from pathology reports following primary surgery at the specialist centre or index surgery performed elsewhere. Tumour grade was recorded according to the *FNCLCC* system. Staging was done by using the *AJCC Cancer Staging Manual* from 2010. Patients with metastatic disease (*n* = 59) or unknown metastatic status at time of diagnosis (*n* = 11) were excluded from time-to-event analyses, resulting in 658 eligible patients.

All patients were discussed in multidisciplinary team meetings and underwent primary surgery or re-resection following UE at one of the three sarcoma centres. Need for plastic, vascular, or endoprosthetic reconstruction at definite surgery was documented. Neoadjuvant, adjuvant, and palliative treatments (RTX, CTX) were recorded.

Follow-up was performed until July 2016. Time to last follow-up or death was calculated from definite surgery to last known date (most recent clinical appointment, telephone contact, or record in obituary column). The study was approved by the institutional review boards at the respective centres.

### Statistical Analysis

Statistical analysis was performed using IBM SPSS statistics version 23.0, Microsoft Excel version 15.19.1 and Stata (Windows version 13.0, Stata Corp., Houston, TX). Continuous variables were summarised as means ± SD, whereas count data were summarised as absolute frequencies with percentages. Associations between two categorical variables, as tumour grade and UE status, were analysed with *χ* ^2^ tests (expected cell counts ≥5) or Fisher’s exact tests (expected cell counts <5). Means between two groups were compared with *t* tests or in case of heteroscedasticity with Wilcoxon’s rank-sum tests. Median survival was estimated with the reverse Kaplan–Meier method by Schemper and Smith. The Kaplan–Meier product-limit estimator was used to calculate survivor functions. Cumulative incidences of LR and distant metastasis (DM) were estimated with competing risk estimators according to Marubini and Valsecchi, treating death-from-any-cause as the competing event of interest. Survivor and cumulative-incidence functions were compared between two or more groups with log-rank and Gray’s tests, respectively. Uni- and multivariable modelling of time-to-event outcomes was performed with Cox-proportional hazards models for the overall survival (OS) endpoint and Fine & Gray’s proportional subdistribution hazards models for the LR and DM endpoints.

A propensity score (PS), defined as the probability of undergoing UE, was estimated for each individual patient according to characteristics at baseline.^8^ The PS was calculated with a binary logistic regression model, including the following variables: gender, patient’s age, anatomical tumour localisation, histologic subtype, tumour depth, size, and tumour grade. Next, an inverse-probability-of-UE weight (IPUEW) was constructed, defined as 1/PS for patients with prior UE and 1/(1−PS) for directly referred patients. Time-to-event analyses for OS, LR, and DM were then weighted using this IPUEW. Sensitivity analyses used a trimmed IPUEW (i.e., the lowest and highest 5% were removed) that did not materially alter the observed associations.

## Results

Patient characteristics at baseline, tumour-related parameters, definite treatment, and postoperative information are presented in Table 1. The mean tumour size was 8.9 cm (range 0.5–47.0 cm; SD: ±6.3 cm), being significantly smaller in superficial STS than in tumours underneath the fascia (6.1 ± 4.8 cm; 10.2 ± 6.4 cm; *t* test, *p* = 0.005). With 457 cases, most STS were located in the lower limbs (62.8%), mainly the thigh. A total of 276 patients had AJCC stage III tumours (40%), and a further 59 had evidence of metastatic disease at time of diagnosis (8.6%).

**Table 1:** Descriptive analysis for all patients and differences depending on prior unplanned excision (UE)

	Total (*n* = 728)	Unplanned excision		Missing	*p* value (*χ* ^2^ test)
		No (*n* = 447)	Yes (*n* = 281)		
Centre					
A	427	262	165	0	0.484
B	98	65	33		
C	203	120	83		
Gender					
Female	352	229	123	0	**0.050**
Male	376	218	158		
Tumour location					
Upper limb	143	73	70	0	**<0.005**
Lower limb	457	310	147		
Trunk	98	50	48		
Other	30	14	16		
Detailed symptoms					
No symptoms	81	54	27	158	0.308
Pain	88	60	28		
Increase	303	202	101		
Pain + increase	96	74	22		
Tumour size (cm)					
0–5	228	83	145	99	**<0.005**
5–10	181	125	56		
>10	220	183	37		
Histology					
Liposarcoma	183	126	57	0	0.270
Myxofibrosarcoma	161	100	61		
Leiomyosarcoma	76	44	32		
Synovial Sarcoma	64	34	30		
MPNST	21	12	9		
Fibrosarcoma	6	3	3		
Other	217	128	89		
Depth					
Superficial	230	93	137	6	**<0.005**
Deep	492	352	140		
Grading					
G1	135	88	47	38	0.298
G2	138	79	59		
G3	417	267	150		
Staging					
IA	35	11	24	38	**<0.005**
IB	98	76	22		
IIA	138	50	88		
IIB	84	59	25		
III	276	198	78		
IV	59	40	19		
Primary LN metastasis					
No	695	430	265	9	0.950
Yes	24	15	9		
Primary distant metastasis					
No	680	412	268	5	0.071
Yes	43	32	11		
Duration of symptoms (mo)					
<6	315	227	88	175	**0.008**
>6	238	136	92		
Amputation					
No	663	399	264	0	**0.031**
Yes	65	48	17		
Plastic reconstruction					
No	505	345	160	3	**<0.005**
Yes	220	101	119		
Vascular reconstruction					
No	687	419	268	3	0.215
Yes	38	27	11		
Endoprosthetic devices					
No	677	409	268	3	**0.022**
Yes	48	37	11		
R-classification					
R0	583	345	238	71	**0.001**
R1	67	52	15		
R2	7	7	0		
Postoperative complications					
No	561	347	214	3	0.730
Yes	164	99	65		
Radiotherapy*					
No	284	184	100	10	0.119
Yes	434	256	178		
Adjuvant radiotherapy					
No	330	216	114	0	**0.041**
Yes	398	231	167		
Chemotherapy*					
No	501	292	209	10	**0.009**
Yes	217	149	68		
Local recurrence					
No	618	384	234	5	0.431
Yes	105	61	44		
Distant metastasis					
No	549	324	225	8	**0.019**
Yes	171	118	53		

Statistically significant results are given in bold

* Comprising neoadjuvant, adjuvant, palliative, or combined treatment regimes

The median duration of symptoms before admission was 6 months (range 3 weeks–30 years). In total, 399 patients had noticed a recent increase of the tumour (70.2%), which was painless in 303 cases and associated with pain in 96. An additional 88 patients had experienced pain only (15.5%), whereas 81 reported no symptoms at all (14.3%).

Altogether, 281 STS patients had undergone UE before referral (38.6%), with a similar incidence at each centre (Table 1). The median delay from UE to definite surgery was 7.9 weeks, with 68.7% of patients treated within 12 weeks (*n* = 193).

At the centres, most patients underwent wide (*n* = 492; 67.6%) and compartmental tumour resection (*n* = 85; 11.7%). Limb-salvage surgery was not feasible in 10.7% of primary surgeries (*n* = 48) compared with 6.0% of re-resections (*n* = 17; *p* = 0.031).

A total of 220 patients required plastic reconstructions, as muscular flaps (35.9%) and split-skin grafts (32.3%). These reconstructions were necessary in 42.7% of re-resections (*n* = 119) compared with 22.6% of planned surgeries (*n* *=* 101; *p* < 0.005). At definite surgery, clear tumour margins (R-Classification; R0) could be achieved in 583 cases (88.7%), microscopically intralesional margins (R1) in 67 cases (10.2%), and macroscopically intralesional margins (R2) in 7 cases (1.1%).^9^,^10^ Postoperative complications that necessitated revision developed in 164 patients (22.6%; Table 1). The rate of postoperative complications did not significantly differ between UE and non-UE -patients (23.3 vs. 22.1%; *p* = 0.730).

Of 434 patients receiving RTX (60.4%), the vast majority underwent postoperative irradiation of the tumour bed (*n* = 398). Patients who underwent re-resection received adjuvant RTX more often than patients who had planned surgery (59.4 vs. 51.7%; *p* = 0.041).

Altogether, 30.2% of patients (*n* = 217) were administered CTX in a neoadjuvant (*n* = 45), adjuvant (*n* = 70), palliative (*n* = 33), or combined treatment regimen (*n* = 69). After a median follow-up of 5.5 years (25th–75th percentile 3.0–8.4 years), 82 patients were alive with disease (11.3%) and 399 had no evidence of disease (54.8%); 129 patients died of STS (17.7%) and 73 died due to other causes (10%). UE patients accounted for 31% (*n* = 40) and non-UE patients for 69.0% (*n* = 89) of cancer-related deaths.

**Fig. 1:**
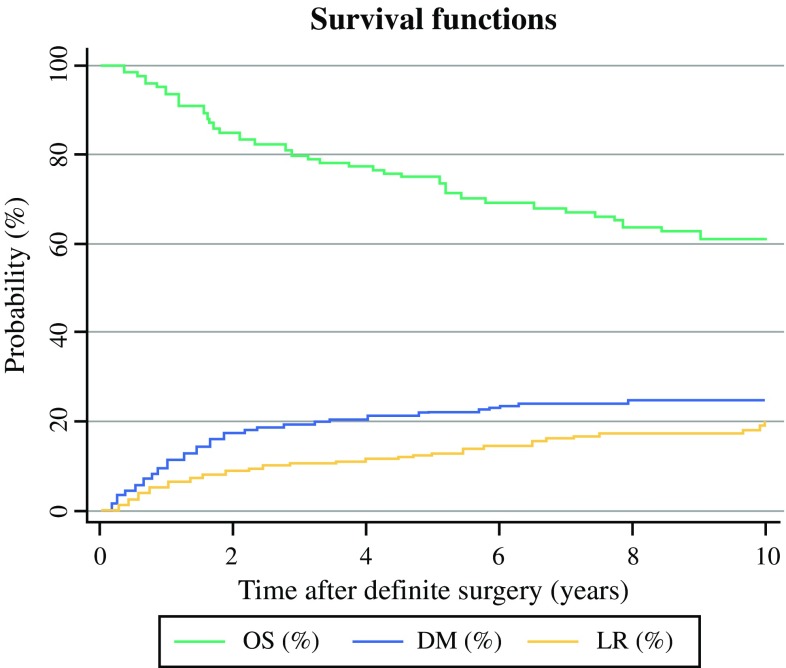
Overall survival (OS) and cumulative incidences of local recurrence (LR) as well as distant metastasis (DM) calculated for patients with localised disease at time of diagnosis (*n* = 658)

Of 658 patients with localised disease at time of diagnosis, 92 subsequently developed LR (14.0%), whereas 137 patients (20.8%) developed DM. The 5- and 10-year OS rates for all patients were 77.7 and 63.8%, respectively. 5 and 10-year risks of LR were estimated at 12.8 and 20.4% and corresponding risks of DM at 22.3 and 25.0% (Fig. 1). Tumour size (*p* = 0.004), grade (*p* = 0.013), stage (*p* < 0.005), histological subtype (*p* = 0.007), symptoms lasting for less than 6 months (*p* < 0.005), and use of CTX (*p* < 0.005) were significant predictors of survival using univariable analysis (Table 2). UE patients undergoing definite surgery later than 12 weeks had a 113% higher relative risk for LR (subdistribution hazard ratio (SHR): 2.135; 95% confidence interval (CI) 1.108–4.112; *p* = 0.023) and a 186% higher relative risk of DM (SHR: 2.863; 95% CI 1.483–5.537; *p* = 0.002) than those treated earlier than 12 weeks.

**Table 2:** Univariable Cox-regression analysis regarding overall survival for patients without primary metastasis at time of diagnosis

	HR	95% CI		*p* value
		Lower	Upper	
Gender (*n* = 658)				
Female	1			0.794
Male	1.043	0.762	1.427	
Tumour location (*n* = 658)				
Upper limb	1			
Lower limb	0.788	0.363	1.711	0.547
Trunk	0.774	0.376	1.592	0.486
Other	0.712	0.301	1.683	0.439
Tumour size (cm) (*n* = 568)				
0–5	1			
5–10	1.953	1.250	3.051	**0.003**
>10	1.953	1.276	2.987	**0.002**
Histology (*n* = 652)				
Fibrosarcoma	NE	NE	NE	NE
Liposarcoma	1			
Myxofibrosarcoma	2.081	1.225	3.536	**0.007**
Leiomyosarcoma	3.510	1.966	6.266	**<0.005**
Synovial sarcoma	2.909	1.548	5.466	**0.001**
MPNST	3.411	1.522	7.648	**0.003**
Other	3.212	1.951	5.289	**<0.005**
Depth (*n* = 652)				
Superficial	1			0.762
Deep	1.055	0.747	1.490	
Grading (*n* = 625)				
G1	1			
G2	2.526	1.218	5.239	**0.013**
G3	5.451	2.854	10.411	**<0.005**
Staging (*n* = 623)				
IA / IB	1			
IIA / IIB	2.846	1.434	5.648	**0.003**
III	6.170	3.214	11.843	**<0.005**
Unplanned excision (*n* = 658)				
No	1			**0.027**
Yes	0.689	0.494	0.961	
Duration of symptoms (mo) (*n* = 492)				
<6	1			**<0.005**
>6	0.538	0.369	0.782	
Postoperative complications (*n* = 654)				
No	1			0.870
Yes	1.030	0.718	1.478	
Radiotherapy* (*n* = 651)				
No	1			0.400
Yes	1.152	0.829	1.600	
Adjuvant radiotherapy (*n* = 657)				
No	1			0.495
Yes	1.118	0.812	1.539	
Chemotherapy* (*n* = 651)				
No	1			**<0.005**
Yes	1.893	1.378	2.601	

Statistically significant results are given in bold

*NE* nonestimable: hazard ratio could not be estimated for the fibrosarcoma-subgroup (*n* = 6, no deaths during follow-up period)

* Comprising neoadjuvant, adjuvant, palliative, or combined treatment regimes

**Fig. 2:**
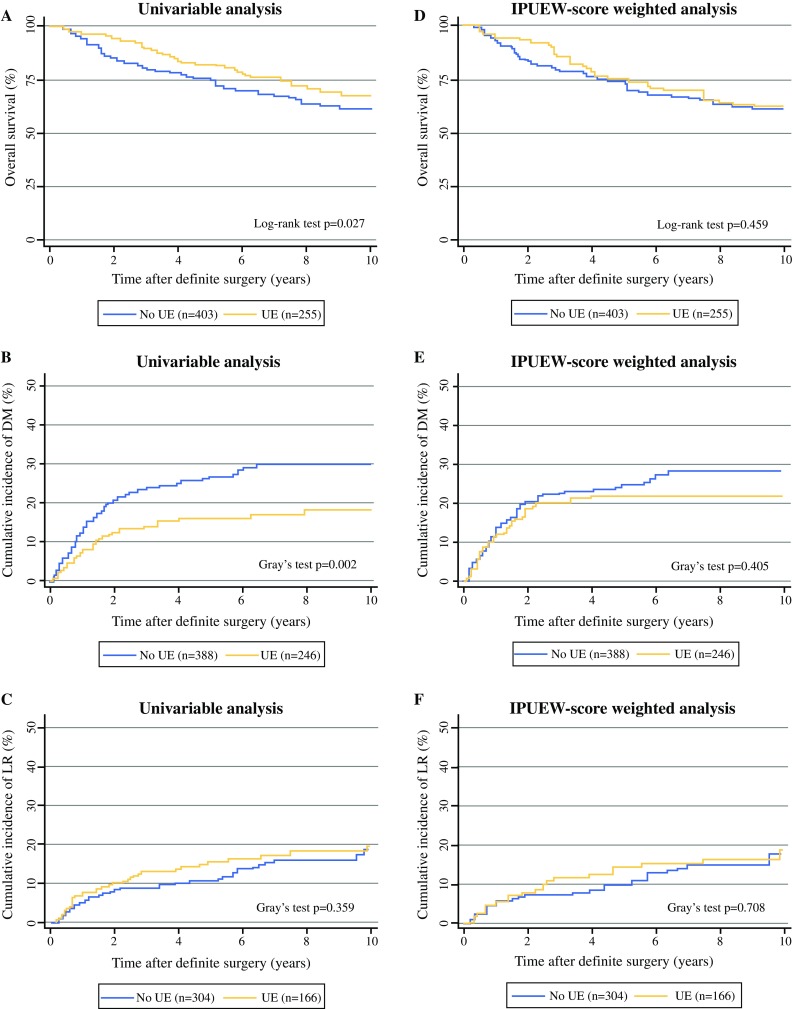
Comparison between patients with prior unplanned excision (UE) and patients undergoing planned surgery (no UE). Juxtaposition of results obtained by univariable analysis and IPUEW-weighted analysis in terms of overall survival (2**a** vs. 2**d**), risk of local recurrence (2**c** vs. 2**f**; LR analysis using a 5% trimmed IPUEW), and risk of distant metastasis (2**b** vs. 2**e**)

Univariable analysis revealed that UEs with consecutive re-resection were significantly associated with improved OS compared with primary surgeries (hazard ratio (HR): 0.689; 95% CI 0.494–0.961; *p* = 0.027; Fig. 2a). At 5 and 10 years, 82.3 and 67.5% of patients with underwent resection were alive compared with 74.8 and 61.4% of patients after planned surgery. UE patients developed DM less frequently than patients with planned surgery (*p* = 0.002; Fig. 2b), whereas LR-free survival rates were similar for both groups (*p* = 0.359; Fig. 2c).

In multivariable analysis, tumour size and grade were confirmed as independent prognostic factors for OS, whereas the favourable “effect” of UE did not prevail after adjustment for selected covariates (HR: 0.737; 95% CI 0.501–1.085; *p* = 0.122; Table 3).

**Table 3:** Multivariable Cox-regression analysis concerning overall survival for patients without primary metastasis at time of diagnosis (*n* = 543)

	HR	95% CI		*p* value
		Lower limit	Upper limit	
Tumour size (cm)				
0–5	1			
5–10	1.864	1.168	2.975	**0.009**
>10	2.309	1.438	3.707	**0.001**
Depth				
Superficial	1			0.266
Deep	0.793	0.527	1.193	
Grading				
G1	1			
G2	2.809	1.337	5.902	**0.006**
G3	5.965	3.066	11.532	**<0.005**
Unplanned excision				
No	1			0.122
Yes	0.737	0.501	1.085	

Statistically significant results are given in bold

However, some significant differences at baseline between patients undergoing UE and those with planned surgery were observed (Table 1). Tumours undergoing UE were significantly smaller (*p* < 0.005), were preferably located superficially (*p* < 0.005), and more often had been noticed by patients some time ago (*p* = 0.008). Male patients were more likely to have their tumours excised inadvertently compared with females (*p* = 0.05). UE patients were younger on average than directly referred ones (56.5 vs. 59.2 years; *p* = 0.036). Because most of the abovementioned parameters are well-established prognostic factors, they potentially have a greater influence on survival than UE itself.

To adjust for these confounding factors, IPUEW analysis was performed as described in the “Methods” section. The odds of being in the UE group differed according to whether tumours were superficially located (odds ratio (OR) for superficial location to being a non-UE patient: 0.29, *p* < 0.0001). After adjusting for the PS, this difference vanished (OR: 1.04, *p* = 0.875), thus supporting the concept that this PS adequately controlled for imbalances between UE- and non-UE patients. After weighting for the IPUEW score, UEs with subsequent re-resection were not significantly associated with an improved survival any more (HR: 0.85; 95% CI 0.56–1.30; *p* = 0.459; Fig. 2d), in conformity with the multivariable analysis. Differences in terms of DM-free survival were likewise lost following IPUEW score weighting (*p* = 0.405; Fig. 2e).

## Discussion

In this retrospective, multicentre study, we analysed the association between unplanned excision, subsequent therapy, local recurrence, distant metastasis, and overall survival in 728 patients with STS. In univariable analysis, patients with prior UE had significantly better overall survival than patients with primary surgery at the tumour centre. However, favourable prognostic factors, such as small, low-grade tumours and superficial location, were more common in patients with UE. These data strongly support the concept that UEs per se, given that they are followed by appropriate definite surgery at a tertiary centre, have no major prognostic impact in patients with STS.

Patients referred following UEs are a widely known phenomenon, accounting for up to 53.3% of all STS patients treated at tumour centres.^3^^–^ 5 In our cohort, 38.6% of patients treated at one of the three centres had undergone UE outwards. The rates that we found are comparable to the 37% reported by Lewis et al. and 34.8% observed by Koulaxouzidis et al.^6^,^11^

A combination of various factors tempts physicians to excise a soft tissue tumour. STS from our cohort undergoing UE had been small and were preferably located clearly visible in the subcutis. Similar observations have been made by other investigators.^3^,^6^,^12^ Interestingly, the quality of symptoms did not significantly alter the rate of inadvertent resections, albeit patients reporting a long history of complaints were more likely to undergo UE. Moreover, we observed a difference for gender and age; significantly more males and younger patients underwent UE.

Due to smaller and more often superficially located tumours, UE patients were less likely to undergo amputation at definite surgery compared with directly referred patients. However, in 42.7% of re-resections after UE, muscular flaps and split-skin grafts were required, whereas plastic reconstructions became necessary in 22.6% of primary surgeries only. Similar disparities have been reported by Potter et al. In their cohort, however, only 5% of planned surgeries but 30% of re-resections necessitated plastic-reconstructive soft tissue coverage.^13^

Because of soft tissue damage, unclear resection margins or frank residual tumour following UE, radical therapeutic approaches are chosen during re-resection in relation to the original tumour size. Whether irradiation following re-resection reduces rate of LR has been discussed controversially. Most authors have concluded that RTX should be administered deliberately, based on tumour extent, postoperative margin status, and histology.^13^^–^ 15 At least in high-grade STS >5 cm with a deep location, the risk for LR seems to be reduced by RTX.^14^ The small average-tumour size notwithstanding, significantly more of our UE patients (59.4%) underwent postoperative irradiation compared with patients who had planned surgery (51.7%). These findings are consistent with previous observations.^13^

Despite more often requiring plastic reconstructions and receiving adjuvant RTX, UE patients did not develop postoperative complications more frequently than non-UE patients in our cohort. There is no doubt, however, that many UE-patients could be protected from disproportionate treatment strategies in relation to the underlying pathology if they would be referred directly.

Preoperative RTX has proven effective in the management of distinct STS subtypes.^16^ However, administration of RTX before definite surgery may not be suitable for UE patients, due to an elevated risk of perioperative complications.^17^

As a result of more complex treatment approaches, UE are associated with considerable financial consequences. According to Alamanda et al., professional costs (i.e., charges billed by surgeons) significantly increased in re-resections compared with primary surgeries.^18^ Even after adjustment for influential parameters, such as tumour grade, site, size, and the fitness of patients before surgery, re-excisions were associated with a 33% increase in professional charges.^18^

Patients subjected to definite surgery at one of the three tumour centres later than 12 weeks following UE had a higher risk for LR and DM compared with those undergoing re-resection shortly after inadvertent resection. Comparable results have been reported by Funovics et al. analysing 310 STS patients with a history of prior UE.^19^

The OS rate of our patients with localised disease at time of diagnosis was 77.7% at 5 years and 63.8% at 10 years. In line with literature, large tumour size and high tumour grade were independent negative prognostic factors.^15^ Several studies have reported an elevated risk for LR despite wide re-resection for prior inadvertently excised STS compared with patients who underwent planned surgery, whereas others did not observe any difference.^5^,^6^,^13^,^20^,^21^

We found that patients who underwent UE with consecutive re-resection had a significantly better prognosis in the univariable analysis (*p* = 0.027). Moreover, they seemed to have a lower risk of developing DM, whereas LR-free survival was comparable to patients treated adequately from the beginning.

After adjusting for the IPUEW score, however, the differences in DM-free survival (*p* = 0.405) and OS (*p* = 0.459) between patients with and without prior UE disappeared. Fiore et al. reported the same conjuncture in a cohort of 597 patients with STS, of whom 53.3% had been referred after UE.^5^ They used a multivariate analysis, including tumour size, depth, grade, and histological subtype, to estimate the effect of UE on OS.^5^ In this study, we also applied multivariable analysis but additionally used IPUEW score-weighting. Both the multivariable adjustment and the IPUEW score-weighted analysis supported our conclusions.

A limitation of this study is its retrospective design, entailing dependency on exhaustive patient history and detailed medical records during follow-up. Additionally, our cohort comprises likewise patients with STS of the extremities, trunk, head, and neck. Moreover, it hardly is possible to assess the impact of potential overtreatment following UE on the patient’s prognosis in a retrospective setting and results apply only to UE patients who have actually been referred to a tertiary centre. Thus, UE patients may have a significantly worse prognosis in case treatment based on the same parameters used for directly referred patients (e.g., tumour size, depth, grade).

## Conclusions

Our study does not support prior findings that unplanned excisions improve patient outcomes. There also is no evidence that inadvertent resections might be harmful to prognosis. Unplanned excisions have, however, an influence on the extent of subsequent therapeutic procedures. More radical surgical approaches requiring plastic reconstruction and the increased need for postoperative radiotherapy compromise the medical condition of patients with prior unplanned excisions. For that reason, they must be avoided by all means.
